# Early and Long-Term Results of 101 Consecutive Patients After Transcarotid Transcatheter Aortic Valve Implantation

**DOI:** 10.3390/jcm15207917

**Published:** 2026-10-14

**Authors:** Damian Hudziak, Monika Gruz-Kwapisz, Radosław Parma, Wojciech Wańha, Sylwia Gładysz-Wańha, Wojciech Wojakowski, Radosław Gocoł

**Affiliations:** 1 Department of Cardiac Surgery, Medical University of Silesia, 40-635 Katowice, Poland; 2 Division of Cardiology and Structural Heart Diseases, Medical University of Silesia, 40-635 Katowice, Poland; radoslaw.parma@gmail.com (R.P.); wojciech.wanha@gmail.com (W.W.);; 3 Department of Electrocardiology and Heart Failure, Medical University of Silesia, 40-635 Katowice, Poland

**Keywords:** transcatheter aortic valve implantation, severe aortic stenosis, transcarotid approach

## Abstract

**Background/Objectives**: Transcatheter aortic valve implantation through the common carotid artery (TC-TAVI) is one of the possible treatment options for patients with ineligible transfemoral approach. The aim of our study was to evaluate the efficacy and safety of patients treated with TC-TAVI in early and long-term follow-up. **Methods**: The study included patients with severe aortic stenosis treated with TC-TAVI. In-hospital outcomes, early, long-term follow-up were presented in accordance with the VARC-3 recommendations. **Results**: The analysis presented 101 patients. We observed high procedural success-97% (*n* = 98). The number of VARC-3 incidents: 3.96% major vascular complications; 0.99% myocardial infarction; 0.99% cardiac tamponade; 0.99% life-threatening bleeding; 3.96% cerebrovascular incidents. The NYHA class decreased significantly compared to the pre-procedure value (2 [1–2] vs. 3 [3–3]; *p* < 0.001). There was a significant improvement in postprocedural cerebral oximetry values compared to baseline on both the implanted and contralateral sides (67.5 [63–71]% vs. 70 [63.5–75]%; *p* < 0.05) and (67 [64–71]% vs. 71 [64–76]%; *p* < 0.05), respectively. The 30-day, 1-year, and 2-year survival rates were 94.1%, 80.2%, and 69.3%, respectively. In the univariate analysis, BAV (HR 4.54; 95% CI: 1.14–17.99; *p* = 0.03) and LVEF ≤ 35% (HR 7.32; 95% CI: 2.59–20.65; *p* < 0.001) were significantly associated with two-year mortality. In an exploratory adjusted Cox regression analysis, BAV (HR 2.63; 95% CI: 1.07–6.45; *p* = 0.03) and LVEF ≤ 35% (HR 3.48; 95% CI: 1.67–7.24; *p* < 0.001) were associated with higher 2-year mortality. **Conclusions:** Our single-center, observational report indicated feasibility and good observed outcomes of the TC-TAVI procedure in an experienced clinical team.

## 1. Introduction

For over two decades, patients with severe aortic stenosis (AS) have been treated with transcatheter aortic valve implantation (TAVI). Numerous randomized trials have confirmed the efficacy of TAVI in high-, intermediate-, and low-risk patients [1,2,3,4,5]. Current guidelines (European Society of Cardiology, European Association for Cardio-Thoracic Surgery, American College of Cardiology, and American Heart Association) recommend the transfemoral artery approach for TAVI (TF-TAVI) [6,7]. However, approximately 5–15% of potential TAVI candidates are still classified as patients with high risk for transfemoral access [8,9].

The aim of our study was to evaluate the efficacy and safety of patients treated with TAVI an alternative-access route via the common carotid artery (TC-TAVI) in early and long-term follow-up at a single academic center.

## 2. Materials and Methods

**Figure 1:**
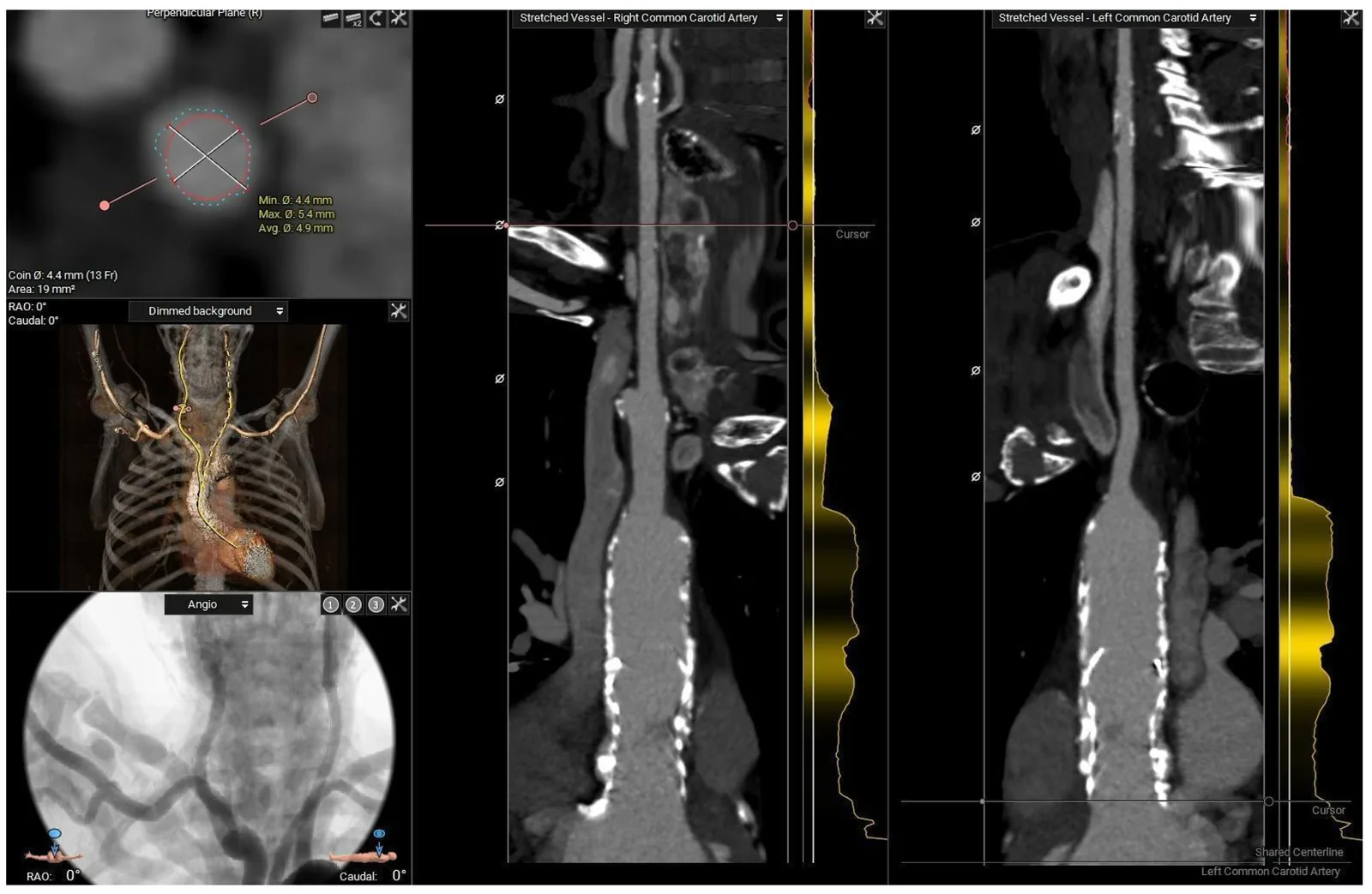
The preprocedural transcarotid approach analysis based on multi-slice computed tomography.

This observational, retrospective, and prospective study included consecutive patients with severe AS treated with TC-TAVI. In-hospital outcomes, early and long-term follow-up were presented in accordance with the Valve Academic Research Consortium–3 (VARC-3) recommendations [10]. According to current recommendations of the European Society of Cardiology and European Association for Cardio-Thoracic Surgery an alternative approach should be considered in patients who do not qualify for surgical aortic valve replacement (SAVR) and TF-TAVI [6,7]. The decision regarding qualification for the TAVI procedure and vascular access was made by the “Heart Team” based on the analysis of multi-slice computed tomography (MSCT) performed using the 3-Mensio software (Pie Medical Imaging, The Netherlands, Version 8.0) (Figure 1).

The diagnostics were always supplemented by transthoracic echocardiography (TTE) if necessary MSCT (the aortic valve calcium score) and coronary angiography. The qualifying condition for TC-TAVI was the inability or high risk of using the femoral access, as confirmed by MSCT. This problem affected patients with iliofemoral artery pathology (critical stenosis, massive calcification and significant tortuosity) and thoracoabdominal aortic diseases (aneurysm, chronic dissection, history of stent-graft implantation, tortuous course, thrombus). MSCT scans of patients eligible for the TC-TAVI procedure had to show no significant calcification and stenosis of the access and contralateral common carotid artery (CCA) >50%. The detailed anatomical criteria for disqualifying patients from transfemoral approach were described in an earlier publication [11]. During the procedure, the following parameters were continuously monitored and measured: electrocardiography, invasive intra-arterial blood pressure, central venous pressure, arterial blood oxygen saturation, and cerebral oximetry (Covidien, Medtronic plc, Dublin, Ireland). The technical aspects of exposure, cannulation, and surgical closure of the carotid artery without clamping have been described in detail in the previous publication in the Surgical Technique section [12]. The MANTA vessel closure device (Teleflex, Morrisville, NC, USA) has been occasionally for off-label use as an alternative to surgical carotid artery closure in cases with a high risk of purse-string suture failure. For rapid pacing, both right and left-ventricular pacing were used. Post-hospital and long-term follow-up were conducted through direct or telephone contact with the patient and their family. Mortality data were obtained from the National PESEL Number Database via the National Cardiac Surgery Registry (KROK). All patients provided written informed consent for the proposed treatment. The local Bioethics Committee waived the requirement for approval of this registry (BNW/NWN/0052/KB/56/26).

### Statistical Analysis

Continuous variables were tested for normality using the Shapiro–Wilk test. As most variables did not follow a normal distribution, they are presented as medians with interquartile ranges (IQRs; 25th–75th percentiles). Categorical variables are expressed as counts and percentages. Comparisons between pre- and postprocedural continuous variables were performed using the Wilcoxon signed-rank test. Categorical variables were compared using the χ^2^ test or Fisher’s exact test, as appropriate. Survival was estimated using the Kaplan–Meier method. Univariable analysis between candidate variables and all-cause mortality during the 2-year follow-up were assessed using Cox proportional hazards regression and are reported as hazard ratios (HRs) with 95% confidence intervals (CIs). Multivariable Cox regression was restricted to a parsimonious, clinically selected set of covariates. Covariates were selected a priori based on clinical relevance and biological plausibility. The multivariable analysis was considered exploratory and hypothesis-generating. All statistical analyses were performed using SigmaPlot version 12.5 (Systat Software, San Jose, CA, USA) and IBM SPSS version 26.0 (IBM Corp., Armonk, NY, USA). A two-sided *p*-value < 0.05 was considered statistically significant.

## 3. Results

Between 2017 and 2023, in the Upper-Silesian Medical Center of the Medical University of Silesia in Katowice, Poland, 1011 TAVI procedures were performed. Approximately 90% were performed via the femoral artery, but 10% (*n* = 101) required an alternative approach.

### 3.1. Study Population

The characteristics of the patients are presented in Table 1.

The median (IQR) age was 77 (72–82) years, and 65% (*n* = 66) were male. Severe aortic valve stenosis was confirmed by clinical symptoms (median NYHA class 3 [3–3]) and echocardiographic parameters. The median surgical risk assessed by EuroSCORE II was 5.4% (4.2–7.4). The current cohort of patients represents the largest studied population after TC-TAVI with long-term follow-up in Poland.

### 3.2. Periprocedural Data

Perioperative characteristics of the study group, the type and size of prosthetic valves are presented in Table 2.

99.1% (*n* = 100) of TC-TAVIs were performed through the left common carotid artery (LCCA). General anesthesia with endotracheal intubation was used in 98% (*n* = 99) of cases. In the presented group, 71.2% of the implanted prostheses were balloon-expandable (BE) valves, Edwards-Sapien 3 (Edwards Lifesciences Corp., Irvine, CA, USA). Self-expanding (SE) valves, CoreValve Evolute R (Medtronic, Minneapolis, MN, USA), and Portico (Abbott Vascular, Santa Clara, CA, USA), accounted for 27.7% and 0.99%, respectively. Rapid pacing was required during 91 TC-TAVI. Of 91 patients who had rapid pacing, left-ventricular pacing was used more frequently than right-ventricular pacing (59% vs. 41%). We observed high procedural success, 97% (*n* = 98), and low procedural mortality, 1.98% (*n* = 2). The first death was caused by aortic annulus rupture after balloon predilation, and the second patient died due to occlusion of the right coronary artery ostium by the native aortic valve leaflet. During one procedure (0.9%), a temporary problem with rapid pacing led to dislocation of the balloon-expandable prosthetic valve.

### 3.3. Postoperative Results

Postoperative data are included in Table 3.

Major vascular complications (VCs) were reported in 3.96% (*n* = 4) of cases, however, damage to the CCA requiring the surgical intervention was only 0.99% (*n* = 1). In 93.1% (*n* = 94) of procedures, the carotid artery was surgically closed with two purse-string sutures after removal of the delivery system. In the remaining cases (6.9%), due to the palpable atherosclerotic plaques and the associated high risk of ineffective hemostasis using standard purse-string sutures, the MANTA vessel closure device was used with good results. Procedural neurological complications were observed in 3.96% of patients (one stroke and three transient ischemic attacks) (Table 3). New onset of atrial fibrillation occurred in 2.97% (*n* = 3) and complete heart block requiring permanent pacemaker (PPM) implantation in 8.9% (*n* = 9). No wound healing problems were observed in any patient treated with TC-TAVI.

### 3.4. Echocardiographic and Cerebral Oximetry Follow-Up

Preprocedural echocardiography was performed in all of 101 patients who were qualified to the implantation, but postprocedural echo in 99 (2 deaths during procedure). After valve implantation, the NYHA class decreased significantly compared to the pre-procedure value (2 [1–2] vs. 3 [3–3]; *p* < 0.001). Follow-up echocardiography confirmed a significant reduction in PG max, Pg mean, Vmax, no difference in left-ventricular ejection fraction (LVEF) and an acceptable (8.9%) rate of moderate and severe paravalvular leak (PVL) (Table 4). In the group of patients with LVEF ≤ 35%, a statistically significant improvement in left-ventricular ejection fraction was observed compared to the baseline. The median LVEF increased from 30% (28–35) to 35% (30–40) after the procedure (*p* < 0.05). Follow-up echocardiography was performed on the day of hospital discharge.

There was a significant improvement in postprocedural cerebral oximetry values compared to baseline on both the implanted and contralateral sides (67.5 [63–71]% vs. 70 [63.5–75]%; *p* < 0.05) and (67 [64–71]% vs. 71 [64–76]%; *p* < 0.05) respectively (Table 4). Additionally, pre- and post-procedure oximetry values did not differ significantly between the access and contralateral CCA (67.5 [63–71]% vs. 67 [64–71]%; *p* = 0.96 and 70 [63.5–75]% vs. 71 [64–76]%; *p* = 0.96), respectively.

### 3.5. Long-Term Results

Data from the PESEL database concerned 101 patients. Information from phone contacts was obtained up till 11 December 2025 (2-years observational time). It was impossible to get in touch with six patients (or their families).

**Figure 2:**
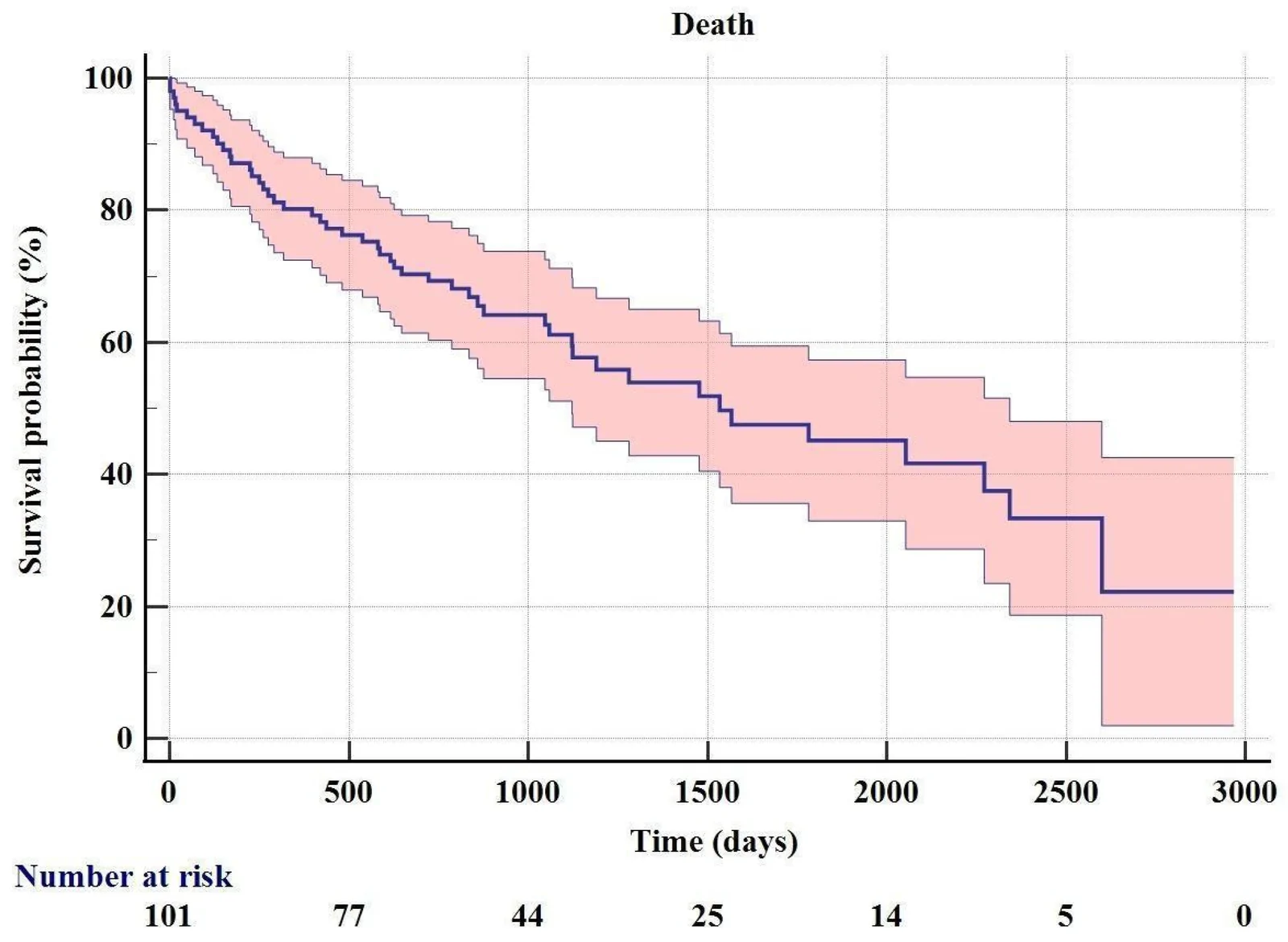
Kaplan–Meier survival analysis.

Seven patients (6.9%) developed neurological complications within 1 year of the TC-TAVI procedure, including 1.98% (*n* = 2) ischemic strokes and 0.99% (*n* = 1) traumatic intracerebral hemorrhage (hospitalization after implantation). Re-hospitalization occurred in 13.8% of patients (4.95% cardiovascular and 8.9% non-cardiovascular). The 30-day, 1-year, and 2-year survival rates were 94.1%, 80.2%, and 69.3%, respectively (Table 3) (Figure 2).

In the univariate analysis, bicuspid aortic valve (BAV) (HR 4.54; 95% CI: 1.14–17.99; *p* = 0.03) and LVEF ≤ 35% (HR 7.32; 95% CI: 2.59–20.6; *p* < 0.001) were significantly associated with two-year mortality after TAVI (Table 5).

In an exploratory adjusted Cox regression analysis BAV (HR 2.63; 95% CI: 1.07–6.45; *p* = 0.03) and LVEF ≤ 35% (HR 3.48; 95% CI: 1.67–7.24; *p* < 0.001) remain associated with higher 2-year mortality. (Table 6).

## 4. Discussion

Improvements in procedural technique, growing operator experience, and new device designs have improved periprocedural and long-term outcomes of TAVI. TC-TAVI accounts for 5-10% of all TAVI procedures, as confirmed by our center’s results (10%) [13,14]. The significant clinical problem after aortic implantation is vascular complications (VCs). The incidence of major VCs in patients undergoing TF-TAVI ranges from 3% to 10% [15,16]. The above results confirm the recent publication in which Eid et al. demonstrated that the prevalence of major VCs was 6.6% among the 2296 patients who underwent TF-TAVI [17]. Results from our analysis have proved a significantly low risk of major VCs after TC-TAVI—3.96% (*n* = 4); however, only one case was directly related to the damage of the CCA. Similar conclusions have been reported in a large meta-analysis, in which researchers noted that patients who underwent TC-TAVI had lower rates of major VCs than after TF-TAVI [18]. Comparing the trans-subclavian (TSc) with the TC approach, Armer et al. reported that TSc access was associated with a numerically higher risk of major VCs (HR 1.65; 95% CI: 0.94–2.89) [19].

On the other hand, some studies reported higher risk of neurological complications after TC-TAVI in comparison with other alternative approaches [19]. Theoretically, higher risk of cerebrovascular (CVA) incidents could be associated with general anesthesia, lower cerebral blood flow, and plaque dislodgement during the procedure. In contrast, a major finding of our report is the low risk of early CVA incidents of 3.96% (1 stroke; 3 transient ischemic attacks). Moreover, the CVA incidence reported in our study is significantly lower than the frequency reported for the TSc and TA approaches by Dahle et al. (6.3%) [20]. Results on the low risk of neurological complications are related to optimal analysis of preprocedural MSCT and an effective technique for closing the carotid artery without clamping (using purse-string sutures). In unpredictable situations localized in the carotid artery area, we used the vascular closure device MANTA. It should be emphasized that in any patient with the vascular closure device (seven patients) we did not observe CVA incidents. Similarly, satisfactory results were demonstrated using the off-label MANTA mechanism in several case series after transaxillary and TSc TAVI by Sacha et al. [21]. It is worth mentioning that this subgroup is small and did not indicate a meaningful conclusion regarding the safety or efficacy of MANTA for carotid closure. This resolution constitutes only a technical observation and an alternative option in patients with palpable atherosclerotic plaques and perceived high risk of ineffective purse-string closure.

In our study, the frequency of neurological complications within 1 year of the TC-TAVI procedure was 7 (6.9%), of which two patients (1.98%) developed ischemic strokes and one (0.99%) traumatic intracerebral hemorrhage. It is worth mentioning that two patients with long-term neurological complications suffered from atrial fibrillation. Similar results were delivered by Usman et al. in their meta-analysis about safety and efficacy of TC-TAVI [22]. On the other hand, Kirker et al. on 3903 patients who underwent TAVI via TC or TSc showed a significantly lower stroke rate in the TC vs. TSc access [23].

Cerebral oximetry is a sensitive tool used for brain perfusion monitoring. The significant improvement in postprocedural cerebral oximetry values compared to baseline on both the implanted and contralateral sides could be associated with increased blood flow through the carotid arteries after valve implantation. Additionally, pre- and post-procedure oximetry values did not differ significantly between the sides (67.5% vs. 67%; *p* = 0.96 and 70% vs. 71%; *p* = 0.96). The above data confirm that the procedure on the carotid artery during TAVI does not disturb the flow through the artery, which translates into a low rate of neurological complications.

The results of large analyses comparing the frequency of permanent pacemaker implantation (PPI) are inconclusive. In our study, we did not report increased risk of new PPI. Complete heart block requiring PPI in our study was demonstrated in 8.9% (*n* = 9) of the cases, so there is no sign of excess PPI in comparison to other alternative approaches [18]. A noteworthy trend towards higher rates of the need for PPI was observed with the TSc approach vs. TC access by Amer et al. [19]. The observed low risk of significant conduction disturbances in TC-TAVI cases is related to high valve implantation and use of BE prosthesis, which in our center constitute 70% of TC implantations. In the French registry comparing the results of the Sapien 3 BE valve with the Evolut R self-expanding valve, 20,918 patients had lower PPM implantation rates after a Sapien 3 BE valve replacement [24], which is in line with our report.

TC-TAVI compared favorably with TF-TAVI in terms of procedural success and perioperative outcomes [25], which supports our findings (Table 3). Follow-up echocardiography (Table 4) in our report confirmed a significant improvement in aortic valve hemodynamics, no impairment of LVEF after replacement, and an acceptable (8.9%) rate of moderate or more PVL. Recent studies have demonstrated shorter hospital stay after TC-TAVI compared with other non-TF alternative approaches (6 vs. 8 days) and a shorter intensive care unit stay, which is comparable to our publication [26]. 

The periprocedural mortality of 1.98% was not related to the vascular access itself. Abraham et al. in their meta-analysis compared TC via transapical approach and reported higher in-hospital mortality after transapical TAVI (8.7% vs. 2.6%), respectively [18]. Another observational study comparing TF-TAVI with TC demonstrated high procedural success with no difference between the two groups (95% vs. 95%) [27]. Similarly, good early clinical outcomes after TC-TAVI vs. TF-TAVI proved Villecourt et al. in propensity-matched analysis [28].

We reported that 30-day and 1-year survival rates were 94.1% and 80.2%, respectively.

Including results from the meta-analysis by Usman et al., our report showed a comparable 30-day mortality (5.3% vs. 5.9%) in the TC-TAVI patients [22]. In turn, Folliguet et al. published similar results about the 1-year survival index (75.8% vs. 80.2% respectively) [29]. Furthermore, during the 2-year follow-up, the survival rate was 69.3%. This higher value is related to the advanced age of patients qualified for aortic replacement and the burden of comorbidities. It should be added that these outcomes reflect the prognosis of elderly, highly comorbid population selected for alternative access and cannot be attributed specifically to the transcarotid approach. It remains unchanged that long-term mortality is associated with baseline comorbidity, frailty, heart failure, coronary disease, renal dysfunction, pulmonary disease, and other competing causes of mortality.

In our report, we show that LVEF ≤ 35% is associated with higher 2-year mortality post-TAVI follow-up. Although aortic valve replacement improves the outcomes, heart failure after implantation is common. Patients with aortic stenosis are exposed to an increase in afterload for years. This remodeling process can begin before severe symptoms and leads to diffuse and focal fibrosis and irreversibility by positive remodeling. On the other hand, coexisting valve diseases or ischemic heart disease may be related to the manifestation of symptoms of heart failure after TAVI.

Contemporary reports have proven that periprocedural and short-term outcomes after TAVI are now largely comparable between patients with BAV and tricuspid aortic valve [30,31]. Our study suggests that, in long-term outcomes, BAV is associated with higher 2-year mortality in the exploratory adjusted analysis post-TAVI follow-up. These findings indicate that differences in the structure and morphology of BAV may be related to distinct hemodynamic changes, thereby modulating patterns of unfavorable left-ventricular remodeling to some extent, which is associated with a worse prognosis. On the other hand, it is more of a hypothesis-generating observation, given the low number of BAV in the cohort and the wide confidence intervals around HR. Larger dedicated studies are needed to evaluate the prognostic relevance of BAV morphology in patients undergoing TC-TAVI.

## 5. Limitations

There are several limitations of this study. It is a single-center registry containing both retrospective and prospective observations. We have no direct or propensity-matched comparator of other non-transfemoral access. There is also a modest selection bias related to anatomy-driven eligibility. The follow-up with telephone visits, ascertainment of non-fatal events and lack of independent event adjudication are also limitations. This finding should be considered hypothesis-generating and requires confirmation in larger cohorts. On the other hand, the study provides a longitudinal assessment of long-term clinical events of patients treated with TC-TAVI.

## 6. Conclusions

In summary, this study demonstrates long-term outcomes of a large group of patients after TC-TAVI in Poland. TC approach constitutes alternative access for patients without treatment option via TF, which is the reason for the lack of randomized trials. Low risk of complications and high effectiveness of the procedure depend on the close cooperation of the Heart Team from qualification to postoperative care. Our single-center observational report indicated feasibility and good observed outcomes of the TC-TAVI procedure. TC-TAVI represents a feasible alternative-access strategy in carefully selected patients unsuitable for transfemoral TAVI. Larger dedicated studies are needed to evaluate the prognostic relevance of BAV morphology in patients undergoing TC-TAVI.

## Figures and Tables

**Table 1:** Patient characteristics.

Variables	TC-TAVI (*n* = 101)
Age (years)	77 (72–82.2)
Male	66 (65.3)
BMI (kg/m^2^)	26.7 (23.5–30.4)
NYHA class	3 (3–3)
EuroSCORE II (%)	5.4 (4.2–7.4)
Prior MI	31 (30.7)
Prior cardiac operation	43 (42.6)
Prior PCI	39 (38.6)
Atrial fibrillation	29 (28.7)
Hypertension	70 (69.3)
BAV	15 (14.8)
LVEF ≤ 35%	20 (19.8)
Diabetes mellitus	71 (70.3)
COPD	28 (27.7)
Thoracic aortic aneurysm	8 (7.9)
PAD	44 (43.6)
Prior stroke	1 (0.99)
Prior TIA	0 (0)
Creatinine level (mg/dL)	1.11 (0.94–1.33)
Hemoglobin level (g/dL)	12.9 (11.5–14.1)

Data are given as the median (interquartile range) or as *n* (%). Abbreviations: BAV: bicuspid aortic valve, BMI: body mass index, COPD: chronic obstructive pulmonary disease, LVEF: left-ventricular ejection fraction, MI: myocardial infarction, NYHA: New York Heart Association, PAD: peripheral arterial disease, PCI: percutaneous coronary intervention, TIA: Transient Ischemic Attack.

**Table 2:** Perioperative outcomes.

Perioperative Outcomes	TC-TAVI (*n* = 101)
Procedural success	98 (97)
Procedural time (min)	75 (65–90)
Prosthesis dislocation	1 (0.99)
General anaesthesia	99 (98)
Right ventricle rapid pacing	37 (36.6)
Left ventricle rapid pacing	54 (53.4)
Valve in valve procedure	0 (0)
Left common carotid artery access	100 (99.1)
Right common carotid artery access	1 (0.99)
Rapid pacing	91 (90)
Balloon aortic valve predilatation	7 (6.9)
Balloon aortic valve postdilatation	11 (10.9)
Coronary occlusion	1 (0.99)
Conversion to SAVR	0 (0)
Procedural mortality	2 (1.98)
MANTA vessel closure device	7 (6.9)
**Types of prosthesis**	
Medtronic Evolute	28 (27.7)
26	10 (9.9)
29	13 (12.8)
34	5 (4.9)
Edwards-Sapien	72 (71.2)
23	27 (26.7)
26	28 (27.7)
29	17 (16.8)
Abbott	1 (0.99)
27	1 (0.99)

Data are given as the median (interquartile range) or as n (%). Abbreviations: SAVR: surgical aortic valve replacement. See Table 1.

**Table 3:** Postoperative outcomes.

Variables	TC-TAVI (*n* = 101)
Myocardial infarction	1 (0.99)
Life-threatening bleeding	1 (0.99)
TIA	3 (3.0)
Stroke	1 (0.99)
Tamponade	1 (0.99)
Major vascular complication	4 (3.96)
Access vascular complication	1 (0.99)
Mechanical ventilation time (minutes)	180 (90–360)
New dialysis	1 (0.99)
Intensive unit care stay (days)	2 (2–2)
Hospital stay (days)	6 (5–7)
Blood transfusion	8 (7.9)
New atrial fibrillation	3 (2.97)
New pacemaker implantation	9 (8.9)
Creatinine level (mg/dL)	1.05 (0.86–1.35)
Hemoglobin level (g/dL)	10.7 (9.82–11.9)
NYHA class	2 (1–2)
Wound infection	0 (0)
Wound hematoma	3 (3.0)
Re-hospitalization	14 (13.8)
Cardiovascular hospitalization	5 (4.95)
Non-cardiovascular hospitalization	9 (8.9)
All-cause 30-day mortality	6 (5.9)
All-cause 1-year mortality	20 (19.8)
1-year neurological complication	7 (6.9)
All-cause 2-years mortality	31 (30.6)

Abbreviations: See Table 1.

**Table 4:** Echocardiographic and cerebral oximetry outcomes.

Variables	Pre-TC-TAVI (*n* = 101)	Post-TC-TAVI (*n* = 99)	*p*-Value
**Echocardiographic parameters**			
LVEF (%)	50 (40–55)	50 (45–55)	0.17
Pg max (mmHg)	70 (57.2–81.5)	19 (14–26)	<0.001
Pg mean (mmHg)	40 (34–48.2)	10 (7–13)	<0.001
AVA (cm^2^)	0.7 (0.6–0.8)	1.9 (1.6–2)	<0.001
Vmax (m/s)	4.2 (3.7–4.6)	2.2 (1.85–2.6)	<0.001
PVL moderate and more	0	9 (8.9)	
**Cerebral Oximetry**			
Implantation side (%)	67.5 (63–71)	70 (63.5–75)	<0.05
Opposite side (%)	67 (64–71)	71 (64–76)	<0.05

Data are given as the median (interquartile range) or as *n* (%). Abbreviations: AVA: aortic valve area, Pg max: aortic valve maximal gradient, Pg mean: aortic valve mean gradient, PVL: paravalvular leak, Vmax: transaortic peak instantaneous velocity.

**Table 5:** Univariable Cox proportional hazards analyses of candidate variables for all-cause mortality during 2-year follow-up after TC-TAVI.

Variable	Hazard Ratio	95% CI	*p*-Value
Arterial hypertension	1.31	0.58–2.97	0.50
Atrial fibrillation	2.08	0.27–15.93	0.47
Diabetes mellitus	0.91	0.38–2.21	0.84
EuroScore > 4 pts	1.11	0.49–2.53	0.79
Age > 80 years	0.84	0.36–1.95	0.69
Tamponade	0.97	0.71–1.32	0.43
Male sex	1.67	0.71–3.93	0.23
BMI < 35	0.88	0.37–2.08	0.78
Previous stroke	-	-	0.43
BAV	4.54	1.14–17.9	0.03
LVEF ≤ 35%	7.32	2.59–20.65	<0.001
Previous MI	1.65	0.70–3.92	0.25

Abbreviations: see Table 1.

**Table 6:** Exploratory multivariable Cox proportional hazards model for all-cause mortality during 2-year follow-up after TC-TAVI.

Variable	Hazard Ratio	95% Cl	*p*-Value
BAV	2.63	1.07–6.45	0.03
LVEF ≤ 35%	3.48	1.67–7.24	<0.001

Variables not included in the model: Arterial hypertension; Diabetes mellitus; Atrial Fibrillation; Previous stroke; Euroscore > 4%; Age > 80; Tamponade; Male sex; BMI > 25; eGFR (mL/min/1.73 m^2^) < 60; Previous MI. Abbreviations: see Table 1.

## Data Availability

All relevant data have been included in this study. The data generated from this study are available from the corresponding author upon reasonable request.

## References

[1] Mack M.J., Leon M.B., Smith C.R., Miller D.C., Moses J.W., Tuzcu E.M., Webb J.G., Douglas P.S., Anderson W.N., Blackstone E.H. (2015). 5-year outcomes of transcatheter aortic valve replacement or surgical aortic valve replacement for high surgical risk patients with aortic stenosis (PARTNER 1): A randomised controlled trial. Lancet.

[2] Kapadia S.R., Leon M.B., Makkar R.R., Tuzcu E.M., Svensson L.G., Kodali S., Webb J.G., Mack M.J., Douglas P.S., Thourani V.H. (2015). 5-year outcomes of transcatheter aortic valve replacement compared with standard treatment for patients with inoperable aortic stenosis (PARTNER 1): A randomised controlled trial. Lancet.

[3] Thyregod H.G.H., Jørgensen T.H., Ihlemann N., Steinbrüchel D.A., Nissen H., Kjeldsen B.J., Petursson P., De Backer O., Olsen P.S., Søndergaard L. (2024). 10-year outcomes of the NOTION trial. Eur. Heart J..

[4] Forrest J.K., Deeb G.M., Yakubov S.J., Gada H., Mumtaz M.A., Ramlawi B., Bajwa T., Teirstein P.S., Tchétché D., Huang J. (2023). 4-year outcomes of patients with aortic stenosis in the Evolut Low Risk Trial. J. Am. Coll. Cardiol..

[5] Braghiroli J., Kapoor K., Thielhelm T.P., Ferreira T., Cohen M.G. (2020). Transcatheter aortic valve replacement in low risk patients: A review of PARTNER 3 and Evolut low risk trials. Cardiovasc. Diagn. Ther..

[6] Praz F., Borger M.A., Lanz J., Marin-Cuartas M., Abreu A., Adamo M., Marsan N.A., Barili F., Bonaros N., Cosyns B. (2025). 2025 ESC/EACTS Guidelines for the management of valvular heart disease. Eur. Heart J..

[7] Otto C.M., Nishimura R.A., Bonow R.O., Carabello B.A., Erwin J.P., Gentile F., Jneid H., Krieger E.V., Mack M., McLeod C. (2021). 2020 ACC/AHA Guideline for the Management of Patients with Valvular Heart Disease: Executive Summary: A Report of the American College of Cardiology/American Heart Association Joint Committee on Clinical Practice Guidelines. Circulation.

[8] Rogers T., Gai J., Torguson R., Okubagzi P.G., Shults C., Ben-Dor I., Satler L.F., Waksman R. (2018). Predicted magnitude of alternate access in the contemporary transcatheter aortic valve replacement era. Catheter. Cardiovasc. Interv..

[9] Beurtheret S., Karam N., Resseguier N., Houel R., Modine T., Folliguet T., Chamandi C., Com O., Gelisse R., Bille J. (2019). Femoral Versus Nonfemoral Peripheral Access for Transcatheter Aortic Valve Replacement. J. Am. Coll. Cardiol..

[10] Généreux P., Piazza N., Alu M.C., Nazif T., Hahn R.T., Pibarot P., Bax J.J., Leipsic J.A., Blanke P., VARC-3 Writing Committee (2021). Valve Academic Research Consortium 3: Updated endpoint definitions for aortic valve clinical research. Eur. Heart J..

[11] Hudziak D., Nowak A., Gocoł R., Parma R., Ciosek J., Ochała A., Deja M., Wojakowski W. (2019). Prospective registry on cerebral oximetry-guided transcarotid TAVI in patients with moderate-high risk aortic stenosis. Minerva Cardioangiol..

[12] Gocoł R., Wojakowski W., Parma R., Darocha T., Hudziak D. (2025). Dissection of the left common carotid artery during a TAVI procedure successfully treated by surgical intervention. Kardiol. Pol..

[13] Wilimski R., Huczek Z., Grodecki K., Kochman J., Rymuza B., Wojakowski W., Hudziak D., Jagielak D., Sacha J., Grygier M. (2023). Nationwide experience with transcarotid transcatheter aortic valve implantation: Insights from the POL-CAROTID registry. Kardiol. Pol..

[14] Hudziak D., Targoński R., Wańha W., Gocoł R., Hajder A., Parma R., Figatowski T., Darocha T., Deja M.A., Wojakowski W. (2023). Comparison of transcarotid versus transapical transcatheter aortic valve implantation outcomes in patients with severe aortic stenosis and contraindications for transfemoral access. Cardiol. J..

[15] Langouet Q., Martinez R., Saint-Etienne C., Soulami R.B., Harmouche M., Aupart M., Le Breton H., Verhoye J.-P., Bourguignon T. (2020). Incidence, predictors, impact, and treatment of vascular complications after transcatheter aortic valve implantation in a modern prospective cohort under real conditions. J. Vasc. Surg..

[16] Raju S., Eisenberg N., Montbriand J., Cusimano R.J., Feindel C., Ouzounian M., Horlick E., Osten M., Tsang W., Roche-Nagle G. (2019). Vascular Complications and Procedures Following Transcatheter Aortic Valve Implantation. Eur. J. Vasc. Endovasc. Surg..

[17] Eid M., Ducroix A., Dang Van S., Ammi M., Rineau E., Binuani P., Bénard T., Delépine S., Chausseret L., Rouleau F. (2026). Major Vascular Complications After Percutaneous Transfemoral Approach for Transcatheter Aortic Valve Implantation in Propensity-Matched Cohort: Impact on Clinical Outcomes and Survival. Eur. J. Cardiothorac. Surg..

[18] Abraham B., Sous M., Sedhom R., Megaly M., Roman S., Sweeney J., Alkhouli M., Pollak P., El Sabbagh A., Garcia S. (2023). Meta-Analysis on Transcarotid Versus Transfemoral and Other Alternate Accesses for Transcatheter Aortic Valve Implantation. Am. J. Cardiol..

[19] Amer M.R., Mosleh W., Megaly M., Shah T., Ooi Y.S., McKay R.G. (2021). Outcomes of Transcarotid Versus Trans-Subclavian Transcatheter Aortic Valve Replacement: A Systematic Review and Meta-Analysis. Cardiovasc. Revascularization Med..

[20] Dahle T.G., Kaneko T., McCabe J.M. (2019). Outcomes Following Subclavian and Axillary Artery Access for Transcatheter Aortic Valve Replacement: Society of the Thoracic Surgeons/American College of Cardiology TVT Registry Report. JACC Cardiovasc. Interv..

[21] Sacha J., Krawczyk K., Gwóźdź W., Bugajski J., Perkowski T., Hobot J., Gierlotka M. (2020). Fully Percutaneous Transaxillary Aortic Valve Replacement with Effective Bailout Plan for Vascular Complications. JACC Cardiovasc. Interv..

[22] Usman M.S., Rawasia W.F., Siddiqi T.J., Mujeeb F.A., Nadeem S., Alkhouli M. (2019). Meta-analysis Evaluating the Safety and Efficacy of Transcarotid Transcatheter Aortic Valve Implantation. Am. J. Cardiol..

[23] Kirker E., Korngold E., Hodson R.W., Jones B.M., McKay R., Cheema M., Heimansohn D., Moainie S., Hermiller J., Chatriwalla A. (2020). Transcarotid Versus Subclavian/Axillary Access for Transcatheter Aortic Valve Replacement with SAPIEN 3. Ann. Thorac. Surg..

[24] Deharo P., Bisson A., Herbert J., Lacour T., Saint Etienne C., Grammatico-Guillon L., Porto A., Collart F., Bourguignon T., Cuisset T. (2020). Impact of Sapien 3 Balloon-Expandable Versus Evolut R self-expandable transcatheter aortic valve implantation in patients with aortic stenosis: Data from a nationwide analysis. Circulation.

[25] Watanabe M., Takahashi S., Yamaoka H., Sueda T., Piperata A., Zirphile X., Leroux L., Peltan J., Labrousse L. (2018). Comparison of Transcarotid vs. Transfemoral Transcatheter Aortic Valve Implantation. Circ. J..

[26] Stańska A., Fijałkowska J., Targoński R., Fijałkowski M., Jaguszewski M., Rogowski J., Szurowska E., Jagielak D. (2020). Transcatheter aortic valve implantation through a transcarotid approach and cerebral injury. Kardiol. Pol..

[27] Hudziak D., Wojakowski W., Malinowski M., Gocoł R., Żak A., Morkisz Ł., Ochała A., Parma R., Smolka G., Ciosek J. (2021). Comparison of the short-term safety and efficacy of transcarotid and transfemoral access routes for transcatheter aortic valve implantation. Kardiol. Pol..

[28] Villecourt A., Faroux L., Muneaux A., Tassan-Mangina S., Heroguelle V., Poncet A., Nazeyrollas P., Ruggieri V.G., Metz D. (2020). Comparison of clinical outcomes after transcarotid and transsubclavian versus transfemoral transcatheter aortic valve implantation: A propensity-matched analysis. Arch. Cardiovasc. Dis..

[29] Folliguet T.A., Teiger E., Beurtheret S., Modine T., Lefevre T., Van Belle E., Gilard M., Eltchaninoff H., Koning R., Iung B. (2019). Carotid versus femoral access for transcatheter aortic valve implantation: A propensity score inverse probability weighting study. Eur. J. Cardio-Thorac. Surg..

[30] Tang Y., Feng Y., Qiao X.C., Yan M., Yin Y., Li W., Xiong T., Li Y., Li J., Bai L. (2026). Outcomes of Transcatheter Aortic Valve Replacement in Mixed Aortic Valve Disease: A Comparison Between Bicuspid and Tricuspid Valves. J. Am. Heart Assoc..

[31] Deeb G.M., Reardon M.J., Ramlawi B., Yakubov S.J., Chetcuti S.J., Kleiman N.S., Mangi A.A., Zahr F., Song H.K., Gada H. (2022). Propensity-Matched 1-Year Outcomes Following Transcatheter Aortic Valve Replacement in Low-Risk Bicuspid and Tricuspid Patients. JACC Cardiovasc. Interv..

